# Oxidation of DNA and RNA in young patients with newly diagnosed bipolar disorder and relatives

**DOI:** 10.1038/s41398-024-02772-8

**Published:** 2024-02-08

**Authors:** Klara Coello, Ilari Jaakko Olavi Mäkinen, Hanne Lie Kjærstad, Maria Faurholt-Jepsen, Kamilla Woznica Miskowiak, Henrik Enghusen Poulsen, Maj Vinberg, Lars Vedel Kessing

**Affiliations:** 1 Copenhagen Affective Disorders Research Centre (CADIC), Psychiatric Center Copenhagen, Copenhagen University Hospital Frederiksberg, Copenhagen, Denmark; 2 University of Copenhagen, Department of Clinical Medicine, University of Copenhagen, Copenhagen, Denmark; 3 Department of Endocrinology, Copenhagen University Hospital Bispebjerg Frederiksberg, Copenhagen, Denmark; 4 Department of Cardiology, Copenhagen University Hospital North Zealand Hillerød, Hillerød, Denmark; 5 Research Unit, Copenhagen University Hospital North Zealand Hillerød, Hillerød, Denmark; 6 Psychiatric Research Unit, Psychiatric Centre North Zealand, Copenhagen University Hospital, Hillerød, Denmark

**Keywords:** Bipolar disorder, Diagnostic markers

## Abstract

Excessive oxidative stress-generated nucleoside damage seems to play a key role in bipolar disorder (BD) and may present a trait phenomenon associated with familial risk and is one of the putative mechanisms explaining accelerated atherosclerosis and premature cardiovascular diseases (CVD) in *younger* patients with BD. However, oxidative stress-generated nucleoside damage has not been studied in young BD patients and their unaffected relatives (UR). Therefore, we compared oxidative stress-generated damage to DNA and RNA in young patients newly diagnosed with BD, UR, and healthy control individuals (HC). Systemic oxidative stress-generated DNA and RNA damage levels were compared by analyzing urinary levels of 8-oxo-7,8-dihydro-2′-deoxyguanosine and 8-oxo-7,8-dihydroguanosine in participants aged 15–25 years, including 133 patients newly diagnosed with BD, 57 UR, and 83 HC. Compared with HC, damage to DNA was 21.8% higher in BD patients (*B* = 1.218, 95% CI = 1.111–1.335, *p* = <0.001) and 22.5% higher in UR (*B* = 1.225, 95% CI = 1.090–1.377, *p* = <0.002), while damage to RNA was 14.8% higher in BD patients (*B* = 1.148, 95% CI = 1.082–1.219, *p* = <0.001) and 14.0% higher in UR (*B* = 1.140, 95% CI = 1.055–1.230, *p* = < 0.001) in models adjusted for sex and age after correction for multiple comparison. Levels did not differ between patients with BD and UR. Our findings support higher oxidative stress-generated nucleoside damage being a trait phenomenon in BD associated with familial risk and highlight the importance of early diagnosis and treatment to prevent illness progression and development of premature CVD.

## Introduction

Bipolar disorder (BD) is a severe, highly heritable mental illness, characterized by recurrent affective episodes and a lifetime prevalence of 1–2% [1]. According to the World Health Organization BD is one of the leading causes of disability worldwide also in young adults [2], and associated with accelerated aging [3] and a shortened life expectancy of 10–12 years in patients aged 15–25 years (Lars Vedel [4]). Natural causes of death by physical disorders are the most prevalent reason for lost life years already from adolescence and increases substantially during early and mid-adulthood, in this way supporting the hypothesis of accelerated aging (L. V. [5]). Cardiovascular diseases are more prevalent in patients with BD compared with the general population (Lars Vedel [6]), and the leading causes of death are also in *young* patients with BD [7]. According to the American Heart Association mood disorders in adolescents are categorized as moderate risk conditions of developing accelerated atherosclerosis and premature cardiovascular diseases [8], with excessive oxidative stress being one of the putative pathophysiological mechanisms explaining the overlap in pathophysiology between CVD and BD [8–10].

Systemic oxidative stress can be measured as oxidative stress-generated nucleoside damage in urine, more specifically as the DNA damage marker; 8-oxo-7,8-dihydro-2′-deoxyguanosine (8-oxodG) and the RNA damage marker; 8-oxo7,8-dihydroguanosine (8-oxoGuo) [11, 12]. Putative pathways leading to accelerated aging and the reduced life expectancy in BD by oxidative stress-generated nucleoside damage include mechanisms of telomere attrition and reduced mitochondrial DNA -copy numbers [13, 14]. Further, excessive oxidative stress-generated nucleoside damage has been found to predict 5-year mortality in elderly patients with type 2 diabetes [15] and may contribute to the overlapping pathophysiology of cardiovascular diseases and BD, as supported by recent findings from our group [10].

Recently, a large meta-analysis investigated oxidative stress-generated nucleoside damage in psychiatric disorders (*n* = 10.151) compared with HC (*n* = 10.532) and found higher oxidative stress-generated nucleoside damage levels in adults with psychiatric disorders in general, as well as in BD, compared with HC [16]. Another newly published meta-analysis of oxidative stress-generated DNA damage included 12 studies, consisting of 808 patients with BD (390 in euthymia, 156 in mania, 137 in depression, 16 in mixed episode, 109 not specified) and 563 HC, found higher levels of DNA damage in depression, but not in manic and mixed states, compared with HC [17], whereas a study from our own group found higher RNA damage levels during (hypo)mania [18]. In a prior investigation partly overlapping with the present study, including adult participants aged 18–70 years, we found persistent higher oxidative stress-generated nucleoside damage levels over time in patients newly diagnosed with BD and in their unaffected relatives (UR) compared with HC, whereas oxidative stress-generated nucleoside damage levels in patients with BD and UR were comparable [19], supporting higher oxidative stress-generated nucleoside damage being a trait phenomenon in BD. Further, excessive oxidative stress-generated nucleoside damage could reflect a risk marker of developing BD in UR. Notably, the present cohort is partly overlapping with our previous cohort [19], however, the median age of participants was notably higher (i.e., 29 years, quartiles: 24–37 years for BD; 27 years, quartiles: 22–35 years for UR; 27 years, quartiles: 24–35 years for HC), than in the present cohort. Studies of oxidative stress-generated nucleoside damage in *young* patients newly diagnosed with BD and their *young* unaffected relatives (UR) are lacking, and highly relevant in the investigation of oxidative stress-generated nucleoside damage as atrait phenomenon in BD.

Therefore, in the present study, we compared oxidative stress-generated nucleoside damage in *young* participant groups of patients newly diagnosed with BD, their UR, and HC individuals, respectively.

We aimed to:

(I) compare oxidative stress-generated nucleoside damage in *young* participant groups of patients newly diagnosed with BD, their UR and HC individuals
(II) compare oxidative stress-generated nucleoside damage in different affective episodes within young patients with BD

We hypothesized that systemic oxidative stress-generated nucleoside damage is:

(I) higher in *young* patients newly diagnosed with BD -and to a lesser degree in their UR- compared with HC
(II) higher in (hypo)manic)/mixed states and depressive states compared with full or partial remission

## Method

### Study design

The present study presents baseline data of the ongoing longitudinal Bipolar Illness Onset Study (BIO) (Lars Vedel [20]). The recruitment took place from June 2015 to December 2020. The BIO-study protocol was approved by the Committee on Health Research Ethics of the Capital Region of Denmark (protocol # H-7–2014–007) and the Danish Data Protection Agency, Capital Region of Copenhagen (RHP2015–023). All participants provided written informed consent, and the study complied with the Declaration of Helsinki principles (Seoul, October 2008).

### Participants

The present study sample partly overlaps with the sample included in prior papers from our group [10, 19, 21], since participants aged <26 years were also included in the present study, whereas the included participants aged 15–17 years have not previously been investigated.

#### Patients newly diagnosed with bipolar disorder

The patients newly diagnosed with BD were recruited at the Copenhagen Affective Disorder Clinic and the Child and Adolescent Mental Health Center Copenhagen, which covers a catchment area of 1.8 million people (the Capital Region). All patients newly diagnosed with BD referred to the two clinics were routinely invited into the BIO study. The inclusion criteria were an ICD-10 diagnosis of BD or a single manic episode and an age between 15–25 years. The exclusion criterion was having an organic BD secondary to brain injury. Patients with BD received treatment as usual at the Copenhagen Affective Disorder Clinic without any involvement of the investigators. Informed consent was obtained from the patient and a guardian for patients under 18 years of age.

#### Unaffected first-degree relatives

After receiving consent from BD patients recruited into the BIO-study, their first-degree UR (siblings and offspring) aged 15–25 were invited to participate with permission from a guardian for patients below 18 years. Exclusion criteria were having a diagnosis of BD, psychotic illnesses, or organic mental disorder. The number of relatives per patient with BD was not restricted.

#### Healthy control individuals

Healthy control individuals aged 15–25 were recruited among blood donors at the Danish Blood Bank at Rigshospitalet, Copenhagen, Denmark, on randomly selected days. The catchment area of the blood bank is identical to that of the Copenhagen Affective Disorder Clinic and the Child and Adolescent Mental Health Center Copenhagen. The exclusion criteria were a personal or first-degree family history of psychiatric disorder that had required treatment.

### Clinical assessment

The initial BD diagnoses were determined by psychiatrists according to ICD-10 and classified as type I or II according to DSM-IV. After obtaining informed consent, PhD students in medicine or psychology confirmed the diagnoses using the semi-structured clinical interview Schedules for Clinical Assessment in Neuropsychiatry (SCAN) [22]. Affective symptoms were rated using the 17-item version of the Hamilton Depression Scale (HAMD-17) [23] and the Young Mania Rating Scale (YMRS) [24]. The Pittsburgh Sleep Quality Index (PSQI) [25] was used to assess the quality of sleep during the past month, and the International Physical Activity Questionnaire (IPAQ) [26] was used to assess the level of physical activity during the preceding week. Daily alcohol intake based on intake the past month, current smoking status and current psychotropic medication use were registered.

### Anthropometric assessment

Height was measured without shoes to the nearest millimeter on a rigid stadiometer. Weight was measured lightly dressed to the nearest 0.1 kilograms on a calibrated digital floor scale (Kern MPE PM).

### Laboratory methods

The urine samples used for measuring 8-oxodG and 8-oxoGuo were collected as part of the longitudinal BIO study, between June 2015 and January 2021. Urine samples were collected between 07.30 and 10.00 AM, following an overnight fast starting at 00.00, using a standard sampling kit without additives (In Vitro, Fredensborg, Denmark). The kit was stored on an ice bed and centrifuged at 4 degrees centigrade, at 1590 × *g*, for a duration of 15 minutes. Afterwards, aliquots of 1.5 ml were transferred to Eppendorf tubes, which were stored at −80 degrees centigrade until 8-oxodG and 8-oxoGuo were assayed. In the Laboratory of Clinical Pharmacology, Rigshospitalet, Copenhagen, Denmark, urine samples were analyzed using ultraperformance liquid chromatography and tandem mass spectrometry (UPLC-MS/MS) [11, 27]. Oxidative stress levels were adjusted to creatinine levels in accordance with Jaffe’s reaction to account for the glomerular filtration rate [28, 29].

### Statistical analyses

Continuous descriptive data were reported as median and interquartile range, whilst descriptive categorical data were reported as numbers and percentage. In our primary analysis, we investigated differences in oxidative stress-generated RNA- and DNA damage between the three participant groups in a linear mixed regression model with the familial relationship as a random factor to account for the interrelatedness of BD and their UR. Three models were run to compare the levels of 8-oxoGuo and 8-oxodG, respectively, between BD, UR, and HC individuals. Firstly, an unadjusted model with oxidative stress level as the dependent variable and group (i.e., BD, UR and HC) as an independent variable. Secondly, in our main model we repeated this model further adjusting for sex and age. Finally, we ran a fully adjusted model adjusting for sex, age, BMI, alcohol intake (units/week) and current smoking status (yes/no).

In analyses within patients, we first conducted linear regression models adjusted for sex and age to compare 8-oxoGuo and 8-oxodG levels, respectively, between different affective states (i.e., ‘(hypo)mania/mania/mixed’, ‘depression’ and ‘full or partial remission’). We pooled hypomanic, manic, and mixed states due to the low prevalence of these phases. Full or partial remission was defined as a HAMD-17 score and a YMRS score <14.

Within patients with BD, we further explored the association between 8-oxoGuo and 8-oxodG levels, respectively, and medication and illness-related variables in separate linear regression analyses adjusted for sex and age, with the following covariates as fixed effect: BD type (I/II), affective state (in a current episode/remission), current psychotropic treatment (yes/no), current lithium treatment (yes/no), current antiepileptic treatment (yes/no), current antidepressant treatment (yes/no), current antipsychotic treatment (yes/no), HAMD-17 total score, YMRS total score, PSQI total score, IPAQ total score, illness duration, and duration of untreated BD. Illness duration was defined as time from first episode (depressive, (hypo)manic or mixed episode) in years, and untreated BD was defined as the time from first (hypo)manic or mixed episode to time of diagnosis in years.

Due to non-normally distributed residuals, 8-oxodG and 8-oxoGuo were transformed to their natural logarithms. Hence, results are expressed as ratios between geometric means of the log-normal distribution. *P* values are presented unadjusted and adjusted for multiple testing using the method of Benjamini and Hochberg, which controls the false discovery rate (FDR) [30], and adjusted *p* values were calculated as defined by Yekutieli and Benjamini [31]. An adjusted *p* value < 0.05 was considered statistically significant. Model check was performed for each individual test to check for any potential bias in variance, and all model assumptions were fulfilled. All statistical analyses were conducted with SPSS version 28 for Windows, (SPSS Inc., Chicago, IL, USA).

## Results

### Demographic and clinical characteristics

The demographic and clinical attributes of the study participants are presented in Table 1. We included 133 young patients newly diagnosed with BD, 57 UR, and 83 HC. The median BMI was within the normal range in the three groups—23.4 (quartiles: 21.5–26.1) in BD patients, 23.4 (quartiles: 20.5–27.5) in UR, and 22.7 (quartiles: 21.4–24.1) in HC. Out of the 133 BD patients, 120 (90.2%) had received their BD diagnosis within the prior two years. The median age of onset in BD patients was 15.0 years (quartiles: 13.0–17.0). The median illness duration was 6.3 years (quartiles: 2.9–9.1), while the median delay in diagnosis (untreated BD) was 3.4 years (quartiles: 1.6–6.4).

**Table 1:** Demographic and clinical variables in patients with newly diagnosed bipolar disorder (BD), their unaffected first-degree relatives (UR) and healthy control individuals (HC).

	BD	UR	HC
*N*	133	57	83
Age	22.6 [20.8–24.3]	22.1 [19.0–23.4]	23.8 [22.1–24.9]
Sex, female	98 (73.7)	30 (52.6)	64 (77.1)
Education (years)	13 [12–15]	13 [11–15]	15 [13–16]
BMI	23.4 [21.5–26.1]	23.4 [20.5–27.5]	22.7 [21.4–24.1]
Waist circumference (cm)	80.0 [72.9–88.8]	80.0 [74.0–90.9]	76.0 [73.0–81.0]
Number of smokers (%)	59 (44.4)	14 (24.6)	14 (16.9)
Alcohol (units per week)	2.0 [0.0–7.0]	2.0 [1.0–6.0]	4.5 [2.0–8.8]
HAMD-17 (total score)	8.0 [4.0–13.5]	1.0 [0.0–3.0]	0.0 [0.0–2.0]
YMRS (total score)	3.0 [1.0–7.5]	1.0 [0.0–2.0]	0.0 [0.0–1.0]
BD I	40 (30.1)		
BD II	89 (66.9)		
BD type not specified	4 (3.0)		
Age of onset (years)	15.0 [13.0–17.0]		
Illness duration^a^ (years)	6.3 [2.9–9.1]		
Untreated bipolar disorder^b^ (years)	3.4 [1.6–6.4]		
Number of affective episodes	10.5 [6.0–19.0]	1 [0–0]	
UR with a psychiatric disorder		4 (7.0)	
Current affective state			
Full or partial remission	82 (61.7)		
Mild/moderate depressive episode	26 (19.5)		
Severe depressive episode	2 (1.5)		
Manic episode	2 (1.5)		
Hypomanic episode	11 (8.3)		
Mixed episode	8 (6.0)		
Not specified	2 (1.5)		
Current psychotropics			
No psychotropic medication	30 (22.6)	55 (96.5)	
Antidepressant	14 (10.5)	1 (1.8)	
Antipsychotic	38 (28.6)	1 (1.8)	
Antiepileptic	61 (45.9)	0 (0)	
Lithium	38 (28.6)	0 (0)	

Continuous variables are presented as median [interquartile range]. Categorical variables are presented as *n* (%).

^a^Illness duration was defined as time from first episode (i.e., depressive, manic, hypomanic, or mixed episode) to time of clinical examination.

^b^Untreated bipolar disorder was defined as time from the first manic, hypomanic, or mixed episode to time of diagnosis.

*BD* bipolar disorder, *UR* unaffected first-degree relative, *HC* healthy control, *BMI* body mass index (kg/m^2^), *IPAQ* International Physical Activity Questionnaires, *PSQI* Pittsburgh Sleep Quality Index, *HAMD-17* Hamilton Depression Rating Scale, *YMRS* Young Mania Rating Scale, *BD I* Bipolar disorder type I, *BD II* Bipolar disorder type II.

Of the patients, 40 (30.1%) had a diagnosis of BD I, whilst 89 (66.9%) had a diagnosis of BD II. Most patients, 82 (61.7%), were in full or partial remission. There were 11 BD patients (8.3%) with more than one UR included in the study sample. Out of 57 UR, 4 (7.0%) had a diagnosis of a psychiatric disorder.

### Association between oxidative stress and participant group

**Fig. 1:**
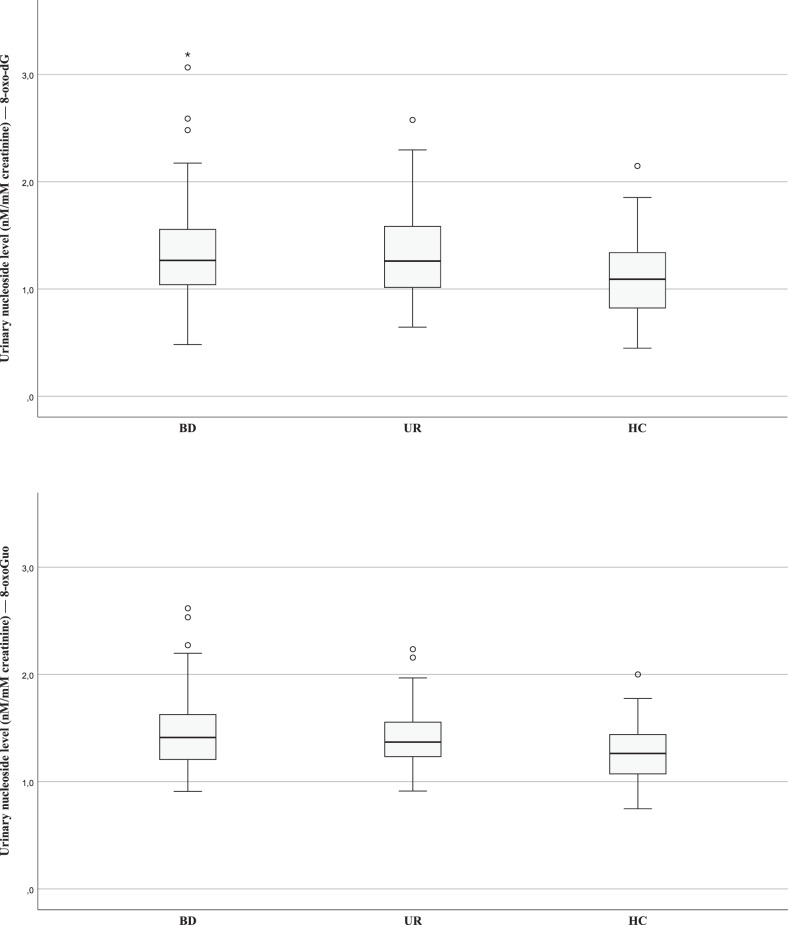
Oxidative stress-generated nucleoside damage in patients newly diagnosed with bipolar disorder (BD), unaffected relatives (UR) and healthy control individuals (HC). Boxplot of oxidative stress marker levels of (A) 8-oxodG (nM/mM creatinine) and (B) 8-oxoGuo (nM/mM creatinine) according to the participant group. The upper and lower hinges represent the third and first quartiles, respectively. The upper and lower whiskers represent the maximum and minimum values, respectively.

Levels of 8-oxodG and 8-oxoGuo in patients newly diagnosed with BD, their UR and HC are illustrated in Fig. 1. The results of comparisons of the levels between participant groups are presented in Table 2.

**Table 2:** Levels of 8-oxo-dG and 8-oxo-Guo in patients with newly diagnosed bipolar disorder (BD), their unaffected first-degree relatives (UR) and healthy control individuals (HC).

	Unadjusted model			Model adjusted for sex and age			Model adjusted for sex, age, BMI, alcohol consumption and smoking		
	Estimate	*p* value	Adjusted *p* value	Estimate	*p* value	Adjusted *p* value	Estimate	*p* value	Adjusted *p* value
8-oxodG									
BD vs HC	1.212 (1.107, 1.326)	0.00004	0.0002	1.218 (1.111, 1.335)	0.00003	0.0002	1.160 (1.050, 1.280)	0.004	0.007
UR vs HC	1.209 (1.081, 1.353)	< 0.001	0.002	1.225 (1.090, 1.377)	< 0.001	0.002	1.188 (1.050, 1.343)	0.006	0.010
BD vs UR	1.002 (0.910, 1.102)	0.974	0.974	0.994 (0.901, 1.096)	0.904	0.957	0.976 (0.880, 1.083)	0.645	0.774
8-oxoGuo									
BD vs HC	1.147 (1.082, 1.217)	0.00001	0.0001	1.148 (1.082, 1.219)	0.00001	0.0001	1.130 (1.058, 1.207)	0.0003	0.001
UR vs HC	1.127 (1.048, 1.213)	0.001	0.003	1.140 (1.055, 1.230)	< 0.001	0.002	1.103 (1.016, 1.198)	0.019	0.029
BD vs UR	1.017 (0.954, 1.085)	0.598	0.769	1.008 (0.944, 1.076)	0.822	0.925	1.023 (0.950, 1.103)	0.533	0.738

Estimates: ratio of geometric means obtained from back transforming the regression coefficients in the linear mixed model. *CI* confidence interval, *BD* bipolar disorder, *UR* unaffected first-degree relative, *HC* healthy control, *BMI* body mass index, *ln* natural logarithm.

In the unadjusted model, 8-oxodG was 21.2% higher in BD compared with HC (*B* = 1.212, 95% CI = 1.107–1.326, *p* = <0.001, adjusted *p* = <0.001) and 20.9% higher in UR, compared with HC (*B* = 1.209, 95% CI = 1.081–1.353, *p* < 0.001, adjusted *p* = 0.002). In the main model adjusted for sex and age, 8-oxodG was 21.8% higher in BD, compared with HC (*B* = 1.218, 95% CI = 1.111–1.335, *p* = <0.001, adjusted *p* = <0.001) and 22.5% higher in UR, compared with HC (*B* = 1.225, 95% CI = 1.090–1.377, *p* < 0.001, adjusted *p* = 0.002). In the fully adjusted model, with adjustment for sex, age, BMI, alcohol consumption and smoking, 8-oxodG was 16.0% higher in BD, compared with HC (*B* = 1.160, 95% CI = 1.050–1.280, *p* = 0.004, adjusted *p* = 0.007) and 18.8% higher in UR, compared with HC (*B* = 1.188, 95% CI = 1.050–1.343, *p* = 0.006, adjusted *p* = 0.010). No differences were found between BD and UR in any of the models.

In the unadjusted model, 8-oxoGuo was 14.7% higher in BD, compared with HC (B = 1.147, 95% CI = 1.082–1.217, *p* = <0.001, adjusted *p* = <0.001) and 12.7% higher in UR, compared with HC (*B* = 1.127, 95% CI = 1.048–1.213, *p* = 0.001, adjusted *p* = 0.003). In the main model adjusted for sex and age, 8-oxoGuo was 14.8% higher in BD, compared with HC (B = 1.148, 95% CI = 1.082–1.219, *p* = <0.001, adjusted *p* = <0.001and 14.0% higher in UR, compared with HC (*B* = 1.140, 95% CI = 1.055–1.230, *p* < 0.001, adjusted *p* = 0.002). In the fully adjusted model, with adjustment for sex, age, BMI, alcohol consumption and smoking, 8-oxoGuo was 13.0% higher in BD, compared with HC (B = 1.130, 95% CI = 1.058–1.207, *p* = <0.001, adjusted *p* = 0.001) and 10.3% higher in UR, compared with HC (*B* = 1.103, 95% CI = 1.016–1.198, *p* = 0.019, adjusted *p* = 0.029). No differences were found between BD and UR in any of the models.

### Association between oxidative stress and affective episodes

The comparisons between levels of nucleoside damage in patients newly diagnosed with BD in different affective states are presented in Table 3.

**Table 3:** Comparison of oxidative stress-generated nucleoside damage between different affective episodes in young patients newly diagnosed with bipolar disorder.

8-oxodG	Estimate	*p* value	Adjusted *p* value	8-oxoGuo	*p* value	Adjusted *p* value
Mania/hypomania/mixed vs remission/partial remission	1.183 (1.009, 1.388)	0.038	0.114	1.084 (0.975, 1.204)	0.134	0.239
Depression vs remission/partial remission	1.033 (0.894, 1.194)	0.660	0.660	0.946 (0.859, 1.041)	0.251	0.301
Mania/hypomania/mixed vs depression	1.146 (0.947, 1.385)	0.159	0.239	1.146 (1.010, 1.301)	0.034	0.114

Estimates: ratio of geometric means obtained from back transforming the regression coefficients in the linear mixed model. *CI* confidence interval. *BD* bipolar disorder, *UR* unaffected first-degree relative, *HC* healthy control, *BMI* body mass index, *ln* natural logarithm

Adjusted for sex and age, 8-oxodG levels were 18.3% higher in ´hypomanic/manic/mixed´ episodes´, compared with partial remission (*B* = 1.183, 95% CI = 1.009–1.388, *p* = 0.038, adjusted *p* = 0.114). 8-oxo-Guo was 14.6% higher in BD patients in ´hypomanic/manic/mixed´ episodes, compared with depressed BD patients (*B* = 1.146, 95% CI = 1.010–1.301, *p* = 0.034, adjusted *p* = 0.114). However, these results did not survive adjustment for multiple comparisons. No differences were found in any of the other comparisons between affective episodes.

### Associations between oxidative stress and illness variables

The associations between 8-oxodG and 8-oxoGuo levels, respectively, and illness variables in BD patients, adjusted for sex and age, are presented in Table 4.

**Table 4:** Associations between oxidative stress-generated nucleoside damage and illness variables in patients newly diagnosed with bipolar disorder.

	8-oxodG			8-oxoGuo		
	Estimate	*p* value	Adjusted *p* value	Estimate	*p* value	Adjusted *p* value
BD type (I vs. II)	1.023 (0.951, 1.101)	0.530	0.904	1.012 (0.962, 1.064)	0.652	0.904
Affective state (episode/remission)	1.096 (0.973, 1.235)	0.128	0.666	1.004 (0.927, 1.088)	0.922	0.934
Receiving psychotropics	0.966 (0.840, 1.111)	0.623	0.904	1.033 (0.942, 1.132)	0.491	0.904
Lithium	1.080 (0.950, 1.229)	0.237	0.880	1.078 (0.991, 1.174)	0.081	0.527
Antiepileptics	0.940 (0.834, 1.059)	0.306	0.884	0.986 (0.911, 0.937)	0.729	0.904
Antidepressants	0.830 (0.689, 1.000)	0.051	0.527	1.019 (0.899, 1.155)	0.766	0.904
Antipsychotics	0.975 (0.857, 1.110)	0.706	0.904	0.991 (0.909, 1.079)	0.829	0.904
HAMD	1.001 (0.992, 1.011)	0.834	0.904	1.000 (0.993, 1.006)	0.934	0.934
YMRS	1.006 (0.995, 1.016)	0.285	0.884	1.006 (0.999, 1.013)	0.071	0.527
PSQI	0.997 (0.981, 1.012)	0.679	0.904	0.999 (0.988, 1.010)	0.833	0.904
IPAQ	1.000 (1.000, 1.000)	0.597	0.904	1.000 (1.000, 1.000)	0.186	0.806
Illness duration^a^	0.998 (0.979, 1.017)	0.812	0.904	1.018 (1.006, 1.031)	0.005	0.130
Untreated BD^b^	1.000 (0.997, 1.004)	0.791	0.904	0.999 (0.997, 1.002)	0.609	0.904

Analyzed separately in models adjusted for sex and age.

Estimates: ratio of geometric means obtained from back transforming the regression coefficients in the linear mixed model. *CI* confidence.

^a^Illness duration was defined as time from first episode (i.e., depressive, manic, hypomanic, or mixed episode) to time of clinical examination.

^b^Untreated bipolar disorder was defined as time from first manic, hypomanic, or mixed episode to time of diagnosis.

*BD* bipolar disorder *UR* unaffected first-degree relative, *HC* healthy control, *HAMD* Hamilton Depression Rating Scale, *YMRS* Young Mania Rating Scale, *PSQI* Pittsburgh Sleep Quality Index, *IPAQ* International Physical Activity Questionnaire.

A positive association was found between 8-oxoGuo levels and illness duration; however, this result did not survive adjustment for multiple comparisons (*B* = 1.018, 95% CI = 1.006–1.031, *p* = 0.005, adjusted *p* = 0.130). There was a trend towards current lithium treatment being positively correlated with 8-oxoGuo (*B* = 1.078, 95% CI = 0.991–1.174, *p* = 0.081, adjusted *p* = 0.527), and current antidepressant use being inversely correlated with 8-oxodG (*B* = 0.830, 95% CI = 0.689–1.000, *p* = 0.051, adjusted *p* = 0.527). After correcting for multiple comparisons, none of these were found significant. No other associations were identified.

## Discussion

This study, for the first time investigated oxidative stress-generated damage on DNA and RNA in a large sample of 133 young patients aged 15–25 years with newly diagnosed BD, 57 of their UR and 83 HC. As hypothesized, we found higher levels of oxidative stress-generated DNA and RNA damage in BD and UR compared with HC, and levels did not differ between BD and UR. Nevertheless, levels of oxidative stress were not found to be enhanced -to a lesser degree- in UR compared with BD, but rather were similarly and to the same level enhanced in UR as in BD compared with HC.

Within patients, oxidative stress-generated RNA damage was higher in´(hypo)manic and mixed´ episodes than during depression and DNA damage higher in ´(hypo)manic and mixed´ episodes than in full or partial remission, however, these results did not persist after adjustment for multiple testing. Similarly, oxidative stress-generated RNA damage was positively associated with illness duration, but not after adjustment of multiple testing. Thus, in models adjusted for multiple comparisons, we did not find statistically significant differences between affective phases in levels of oxidative stress-generated nucleoside damage nor associations with the investigated illness variables.

Our findings of higher levels of oxidative stress-generated damage to DNA and RNA in patients with newly diagnosed BD compared with HC are in line with previous findings from adult studies [17, 32], including several studies from our research group [18, 19, 21, 33].

The higher levels of oxidative stress-generated damage to DNA and RNA in *young* UR compared with HC also concur with previous findings from our research group in the partly overlapping sample of adult participants [19, 21], and support excessive oxidative stress-generated nucleoside damage being a trait phenomenon in BD. This is further supported by our finding of no differences in levels between BD and UR, further suggesting that elevated oxidative stress-generated nucleoside damage is a risk factor for developing BD.

On a more general level, the higher levels of oxidative stress-generated nucleoside damage in UR compared with HC are also in line with results from a recent adult study on BD patients and their first-degree UR, which reported higher serum oxidants and conversely lower serum antioxidants in BD and in UR compared with HC [34].

Child- and adolescent-onset BD is associated with a more severe course of illness than adult-onset BD [35]. However, studies focusing on oxidative stress-generated damage to DNA and RNA in *young* individuals are scarce. To the best of our knowledge, only two small studies have investigated oxidative stress-generated damage in young BD patients and HC. The first study (*n*, BD = 16; *n*, HC = 13) investigated oxidative stress-generated damage to lipids and proteins and, conflicting with our findings, reported lower levels of lipid peroxidation in first-episode BD patients than in HC, while finding no differences in levels of protein carboxylation [36]. Likewise conflicting with our findings, the other study (*n*, BD = 29; *n*, HC = 25) reported no significant differences in lipid peroxidation between young patients with BD and HC [37]. Finally, one study without a control group (*n*, BD = 30) reported lower lipid peroxidation and protein carboxylation in young patients with BD compared with data on adult patients previously published in another study by one of the co-authors [38]. In our own longitudinal study following adult patients newly diagnosed with BD, their UR and HC did not support an increase but rather persistent elevation of oxidative stress-generated nucleoside damage over time [19].

The lack of association between oxidative stress-generated nucleoside damage and illness variables is in accordance with the findings from the larger, partly overlapping adult study sample [21].

The main strength of the present study is that we investigated validated markers of systemic oxidative stress in a scarcely studied area, including 133 *young* patients newly diagnosed with BD, 57 UR and 83 HC individuals in the age range 15–25 years. To our knowledge, the present study is the first to investigate oxidative stress-generated nucleoside damage in *young* patients newly diagnosed with BD, their UR and HC. As a strength, by including UR, oxidative stress-generated nucleoside damage could be investigated as a trait phenomenon in BD. Finally, the study profited from using the golden standard of measuring oxidative stress-generated nucleoside damage [11].

Nevertheless, several limitations apply to the study. Firstly, the modest number of UR may have inhibited the identification of possible true differences in levels of oxidative stress-generated damage to DNA and RNA between patients and UR. Nonetheless, our findings are in line with previous findings from the entire BIO study with a larger UR sample [19, 21]. Notwithstanding, replication of these findings in future studies in *young* patients with BD, UR and HC is warranted.

Secondly, most of the patients were in full or partial remission at baseline, and only 49 (36.8%) patients were in a current episode. The hypomanic, manic, and mixed episodes were pooled together to increase statistical power; however, even though we may still have overseen true differences between affective episodes. Accordingly, we observed a trend of 8-oxodG being higher in (hypo)manic/mixed phases compared with full or partial remission, and 8-oxoGuo higher in (hypo)manic/mixed phases compared with depression, but these associations did not persist after adjustment for multiple comparisons. Thus, our findings regarding affective phase alterations in oxidative stress should be interpreted with caution and future studies with a higher prevalence of affective phases and, consequently, higher statistical power are warranted.

Finally, the cross-sectional design hinders the determination of causality, and thus, our findings can only be considered as associations.

In conclusion, we found higher levels of oxidative stress in *young* patients with newly diagnosed BD and their UR compared with HC, supporting excessive oxidative stress-generated nucleoside damage being a trait phenomenon in BD associated with familial risk. Higher levels of oxidative stress in *young* patients with BD and UR may contribute to the well-known enhanced risk of accelerated atherosclerosis and premature cardiovascular diseases and underscores the importance of early diagnosis and treatment of BD to prevent BD illness progression, accelerated atherosclerosis and premature cardiovascular diseases.

## Data Availability

Data is currently not available as the study is ongoing. Data will be available upon request after the study has ended (estimated last patient in 2027).
